# Response from Co‐Editor‐in‐Chiefs to letter regarding “ALVAC‐fIL2, a feline interleukin‐2 immunomodulator, as a treatment for sarcoids in horses: A pilot study”

**DOI:** 10.1111/jvim.16494

**Published:** 2022-07-20

**Authors:** Stephen P. DiBartola, Kenneth W. Hinchcliff

Pilot studies do not test hypotheses about the effect of an intervention but rather assess the feasibility of an approach to be used in a larger scale study (https://nccih.nih.gov/grants/whatnccihfunds/pilot_studies). A pilot study does not ask “does it work?” but rather, “can it be done?” The typically small sample size of pilot studies precludes formal assessments of safety or tolerability (ie, failure to identify concerns does not mean the intervention is necessarily safe). Likewise, small sample size means such studies are not adequately powered to answer questions about efficacy. The stated objectives of the study by Saba et al were: “to determine tolerability, overall response rate, time to response, and progression‐free survival.” Because pilot studies are not able to achieve such aims, the term “pilot” should not have been used to describe the study. As mentioned in our author guidelines, the Journal makes a concerted effort to avoid inappropriate use of the designation “pilot study,” and has done so for some time. We regret the error in this instance and thank Drs Rishniw and White for drawing it to our attention.

